# The calcineurin inhibitor Sarah (Nebula) exacerbates Aβ42 phenotypes in a *Drosophila* model of Alzheimer's disease

**DOI:** 10.1242/dmm.018069

**Published:** 2016-03-01

**Authors:** Soojin Lee, Se Min Bang, Yoon Ki Hong, Jang Ho Lee, Haemin Jeong, Seung Hwan Park, Quan Feng Liu, Im-Soon Lee, Kyoung Sang Cho

**Affiliations:** 1 Department of Biological Sciences, Konkuk University, Seoul 05029, Republic of Korea; 2 Department of Oriental Medicine, Dongguk University, Gyeogju 38066, Republic of Korea; 3 Department of Oriental Neuropsychiatry, Graduate School of Oriental Medicine, Dongguk University, Gyeonggi 10326, Republic of Korea

**Keywords:** Alzheimer's disease, Amyloid-β42, *Drosophila*, DSCR1 (RCAN1), *sarah* (*nebula*)

## Abstract

Expression of the Down syndrome critical region 1 (DSCR1) protein, an inhibitor of the Ca^2+^-dependent phosphatase calcineurin, is elevated in the brains of individuals with Down syndrome (DS) or Alzheimer's disease (AD). Although increased levels of DSCR1 were often observed to be deleterious to neuronal health, its beneficial effects against AD neuropathology have also been reported, and the roles of DSCR1 on the pathogenesis of AD remain controversial. Here, we investigated the role of *sarah* (*sra*; also known as *nebula*), a *Drosophila DSCR1* ortholog, in amyloid-β42 (Aβ42)-induced neurological phenotypes in *Drosophila*. We detected *sra* expression in the mushroom bodies of the fly brain, which are a center for learning and memory in flies. Moreover, similar to humans with AD, *Aβ42*-expressing flies showed increased Sra levels in the brain, demonstrating that the expression pattern of DSCR1 with regard to AD pathogenesis is conserved in *Drosophila*. Interestingly, overexpression of *sra* using the *UAS-GAL4* system exacerbated the rough-eye phenotype, decreased survival rates and increased neuronal cell death in *Aβ42*-expressing flies, without modulating *Aβ42* expression. Moreover, neuronal overexpression of *sra* in combination with *Aβ42* dramatically reduced both locomotor activity and the adult lifespan of flies, whereas flies with overexpression of *sra* alone showed normal climbing ability, albeit with a slightly reduced lifespan. Similarly, treatment with chemical inhibitors of calcineurin, such as FK506 and cyclosporin A, or knockdown of calcineurin expression by RNA interference (RNAi), exacerbated the *Aβ42-*induced rough-eye phenotype. Furthermore, *sra-*overexpressing flies displayed significantly decreased mitochondrial DNA and ATP levels, as well as increased susceptibility to oxidative stress compared to that of control flies. Taken together, our results demonstrating that *sra* overexpression augments Aβ42 cytotoxicity in *Drosophila* suggest that *DSCR1* upregulation or calcineurin downregulation in the brain might exacerbate Aβ42-associated neuropathogenesis in AD or DS.

## Abstract (precis)

***Drosophila* Collection:** Chronically increased levels of Sarah (Nebula), a calcineurin inhibitor, cause mitochondria dysfunction and subsequently increased *Aβ42*-induced cytotoxicity in *Drosophila*.

## INTRODUCTION

Alzheimer's disease (AD) is a neurodegenerative disorder, with typical clinical symptoms including memory loss and changes in personality, and is characterized by extracellular senile plaques, neurofibrillary tangles, neuronal cell death and progressive neurodegeneration (Hardy and Selkoe, 2002; Walsh and Selkoe, 2004). The extracellular plaques predominantly contain amyloid-beta (Aβ) peptides (Wirths et al., 2004), and important roles of Aβ as a risk factor in the pathogenesis of AD have been suggested (Mattson, 2004; Wirths et al., 2004; Ashe and Zahs, 2010).

Several molecular changes have been identified as downstream events of Aβ accumulation during the development of AD, which include an increase in oxidative stress in the brains of affected individuals (Markesbery, 1997). The formation of Aβ oligomers generates hydrogen peroxide, a source of hydroxyl radicals that initiates membrane lipid peroxidation (Hensley et al., 1994; Bezprozvanny and Mattson, 2008). Mitochondrial abnormalities, such as decreased respiration by mitochondria and increased levels of reactive oxygen species (ROS), are also early pathological characteristics of AD (Maurer et al., 2000; Lin and Beal, 2006). Aβ peptides promote Ca^2+^ influx by forming ion-conducting pores or inducing membrane lipid peroxidation (Bezprozvanny and Mattson, 2008). This disruption of neuronal Ca^2+^ homeostasis is implicated in AD pathogenesis. Moreover, the c-Jun N-terminal kinase (JNK) and extracellular signal-regulated kinase (ERK) pathways are activated in AD brain (Zhu et al., 2002; Pearson et al., 2006) and promote neurodegeneration during AD progression (Mills et al., 1997; Desdouits-Magnen et al., 1998; Bozyczko-Coyne et al., 2001; Borsello and Forloni, 2007). In addition, neuroinflammation is also associated with AD pathology (Akiyama et al., 2000), where inflammation is triggered by Aβ42-activated glial cells, thus inducing proinflammatory cytokines and chemokines, which leads to neurodegeneration, cell death and neuronal dysfunction in the brains of individuals with AD (Ho et al., 2005; Glass et al., 2010; Weitz and Town, 2012).

Several groups have developed AD models in *Drosophila*; they found that overexpression of *Aβ42* leads to locomotive defects, learning and memory dysfunction, neurodegeneration, and a reduced lifespan (Finelli et al., 2004; Greeve et al., 2004; Iijima et al., 2004; Crowther et al., 2005). Additionally, overexpression of *Aβ42* in *Drosophila* neurons induces caspase-dependent apoptosis via hyperactivation of JNK (Hong et al., 2011, 2012) and ERK (Park et al., 2013), as well as increased glial cell proliferation (Park et al., 2013).

Down syndrome (DS) has been reported to be associated with AD (Lott and Head, 2001, 2005). Most individuals aged over 40 years with DS show a neuropathology characteristic of AD (Lott and Head, 2005). Triplication of the amyloid precursor protein (*APP*) and beta-site APP cleaving enzyme 2 (*BACE2*) genes, which are located on chromosome 21, is believed to be responsible for the AD neuropathology observed in the brain of individuals with DS (Lott and Head, 2001). Other DS-related genes might also play a role in AD neuropathology. Among these genes, Down syndrome critical region 1 (*DSCR1*) is extensively associated with AD neuropathology (Harris et al., 2007; Keating et al., 2008; Ermak et al., 2011; Lloret et al., 2011). DSCR1 – also known as regulator of calcineurin 1 (RCAN1), Adapt78 and myocyte-enriched calcineurin interacting protein (MCIP) – is an endogenous inhibitor of calcineurin, a calcium/calmodulin-dependent serine/threonine phosphatase (Kingsbury and Cunningham, 2000; Rothermel et al., 2000; Davies et al., 2007), which is the only neuronal phosphatase regulated by cytosolic Ca^2+^ levels (Baumgärtel and Mansuy, 2012). Dysregulated neuronal Ca^2+^ homeostasis is associated with cellular processes in AD (Bezprozvanny and Mattson, 2008); thus, DSCR1 and its target calcineurin have been implicated in a variety of events that occur in the brains of individuals with AD (Ermak and Davies, 2003; Reese and Taglialatela, 2010; Ermak et al., 2011; Reese et al., 2011). DSCR1 mRNA and protein levels are increased in the brains of individuals with AD (Ermak et al., 2001; Harris et al., 2007), and DSCR1 is associated with neuronal cell death (Sun et al., 2011, 2014; Kim et al., 2013; Wu and Song, 2013). Overexpression of *DSCR1* promotes oxidative-stress- or calcium-overloading-induced apoptosis through caspase-3 activation (Sun et al., 2011, 2014; Wu and Song, 2013). Moreover, *DSCR1* overexpression in mouse models causes hippocampal deficits that alter learning and memory as well as moderate behavioral impairment (Martin et al., 2012; Bhoiwala et al., 2013). However, other studies demonstrate that DSCR1 has a protective effect against calcium-mediated stress-induced damage (Ermak et al., 2012) and oxidative-stress-induced apoptosis (Kim et al., 2013). More recently, a neuroprotective role for DSCR1 has been reported in ischemic brain injury (Brait et al., 2012; Sobrado et al., 2012). Moreover, inhibition of calcineurin ameliorates neurodegenerative and abnormal morphologies, such as dendritic spine loss and dendritic simplification, in *APP*-overexpressing transgenic mouse cells (Wu et al., 2010).

The *Drosophila* genome contains a *DSCR1* ortholog, *sarah* (*sra*; also known as *nebula*) (Chang et al., 2003). When overexpressed, *sra* suppresses the phenotypes induced by the constitutively active calcineurin A subunit (Takeo et al., 2006). This suggests that *sra* inhibitory action against calcineurin is well conserved across species. It has previously been reported that both knockout and overexpression of *sra* causes severe learning defects, mitochondrial dysfunction and increased ROS levels (Chang et al., 2003; Chang and Min, 2005). However, a recent study demonstrated that upregulation of *sra* exerted a neuroprotective effect against *APP*-induced neuronal impairments such as neurodegeneration, over-proliferation of synaptic boutons, axonal transport defects and impaired larval movement, in AD model flies (Shaw and Chang, 2013).

Although DSCR1 is associated with AD, its role in the development of AD remains controversial. Therefore, in the current study, we investigated the role of *sra* in the presence and absence of Aβ42 in *Drosophila*. Interestingly, overexpression of *sra* exacerbated the rough-eye phenotype of *Aβ42*-expressing flies and decreased their survival. The *sra-*overexpressing flies showed decreased mitochondrial DNA (mtDNA) content and increased susceptibility to oxidative stress. These results suggest that chronically increased *sra* levels might cause mitochondria dysfunction and subsequently increase *Aβ42*-induced cytotoxicity.

## RESULTS

### Ectopically expressed *Aβ42* increased *sra* expression levels in *Drosophila* brain

**Fig. 1.**
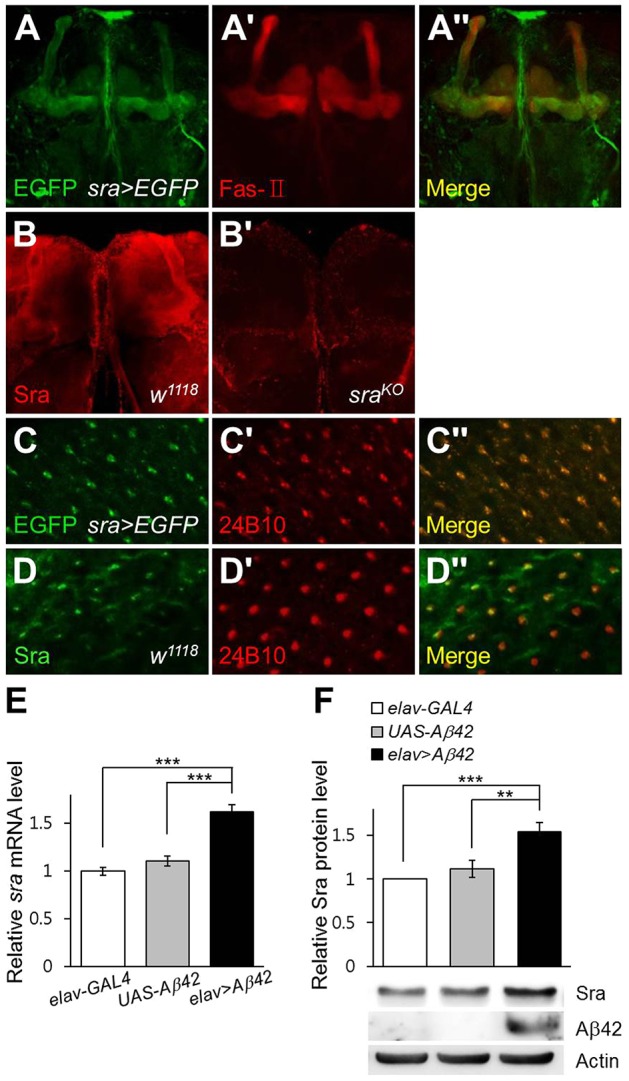
**Intrinsic** ***sra*** **expression is shown in the mushroom bodies and photoreceptor neurons, and is upregulated by *Aβ42* expression.** (A-D) Expression patterns of *sra* were examined using enhanced green fluorescence protein (EGFP) activity and anti-Sra antibody staining in *Drosophila* mushroom bodies (A,A″,B,B′; ×200) and third-instar larval eye imaginal discs (C,C″,D,D″; ×1200). Mushroom bodies and photoreceptor neurons are highlighted by staining with anti-Fas-II (A′,A″; ×200) and anti-Chaoptin (C′,C″,D′,D″; ×1200, 24B10) antibodies, respectively. (E,F) Sra mRNA (E) and protein (F) levels were upregulated in *Aβ42*-expressing flies (*elav*>*Aβ42*) compared with those of the control (*elav-GAL4* or *UAS-Aβ42*). All data are expressed as mean±s.e.m. (E, Tukey–Kramer test, *n*≥6, ***P<0.001; F, Tukey–Kramer test, *n*=10, **P<0.01, ***P<0.001). Fas-II, Fasciclin II.

To estimate the function of *sra* in *Drosophila*, we generated *sra-GAL4* flies, in which *GAL4* expression was controlled by the *sra* promoter, and investigated *sra* promoter activity by crossing *sra-GAL4* with *UAS*-*2×EGFP* flies. Interestingly, *sra* was highly expressed in the mushroom bodies of the brain, which are an important center for learning and memory in *Drosophila*, the region highlighted with an anti-Fasciclin-II antibody (Fig. 1A-A″). Sra expression in mushroom bodies of wild-type and *sra* mutant (*sra^KO^*) flies was confirmed by immunohistochemistry with an anti-Sra antibody (Fig. 1B,B′). Moreover, *sra* promoter activity was also detected in the photoreceptor neurons of the eye imaginal disc, which were highlighted with an anti-Chaoptin antibody (24B10) (Fig. 1C-C″). Sra expression in this tissue was also confirmed with anti-Sra antibody staining (Fig. 1D-D″). These results suggest that *sra* might function in the brain and developing eye.

Because DSCR1 levels are increased in the brains of individuals with AD (Ermak et al., 2001), we investigated whether *sra* is similarly upregulated in *Drosophila* brain by ectopically expressing human *Aβ42*. The *sra* expression levels in fly head regions pan-neuronally expressing human *Aβ42* was measured by real-time quantitative PCR and compared with that of a control. Interestingly, *sra* expression in the head of *Aβ42*-expressing flies was higher than that of the control (Fig. 1E). Consistently, *Aβ42* expression also increased Sra protein levels (Fig. 1F), implying that the function of DSCR1 in Aβ42-induced pathology is conserved in *Drosophila*. By contrast, *APP* overexpression did not affect Sra levels (Shaw and Chang, 2013; Fig. S1), which suggests that the downstream events of *APP* expression are different from those of *Aβ42*.

### Overexpression of *sra* aggravates *Aβ42*-induced neurological phenotypes

**Fig. 2.**
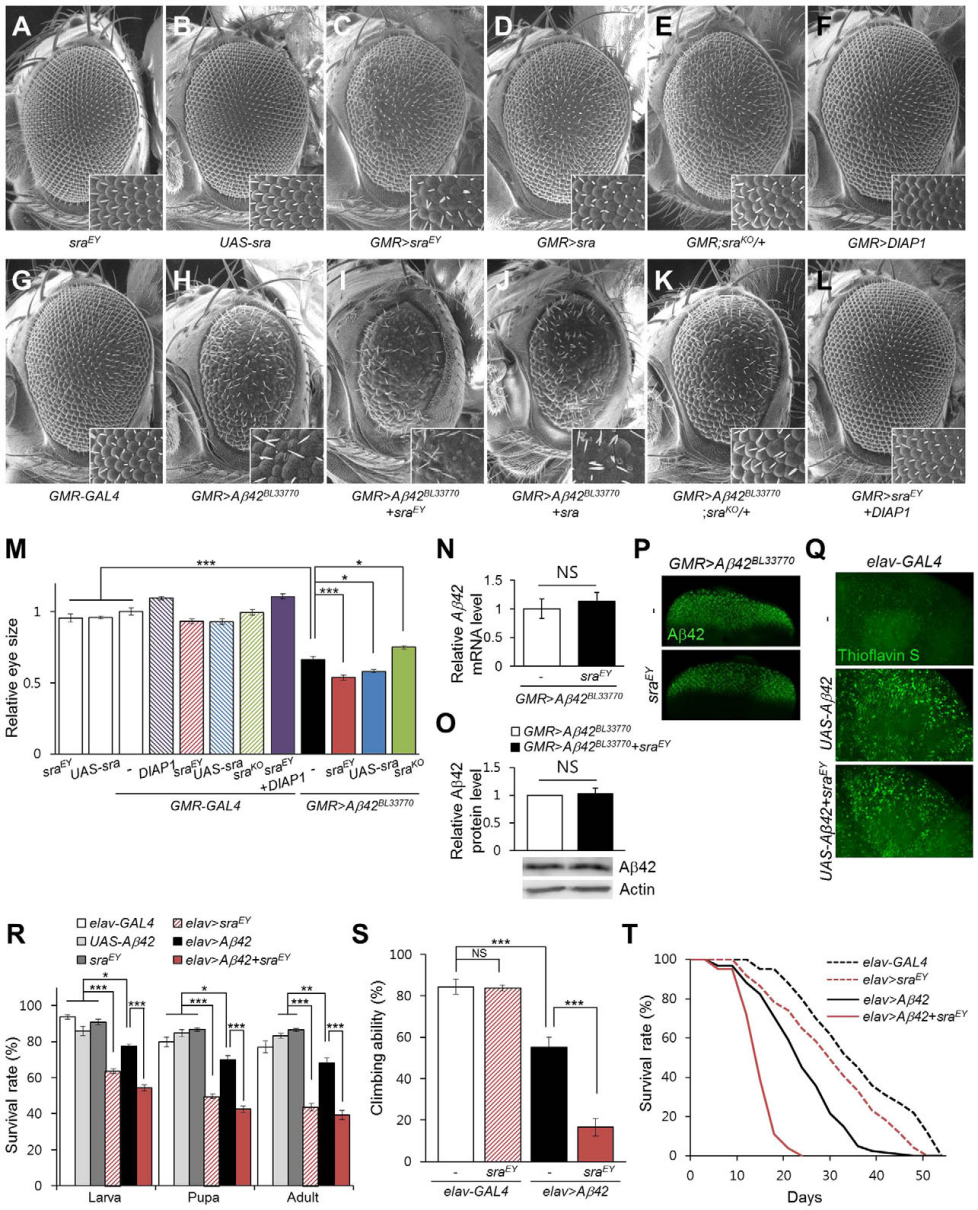
**Overexpression of *sra* exacerbates *Aβ42*-induced phenotypes in *Aβ42*-expressing flies.** (A-M) The eye phenotypes induced by ectopic expression of *Aβ42* in the developing eye were aggravated by *sra* overexpression. The *Aβ42*-expressing adult eye (H) was severely distorted as a result of neurodegeneration when compared with the control eye (A,B,G). Overexpression of *sra* alone resulted in a marginally rough-eye phenotype (C,D) compared with that of the control (A,B,G). Overexpression of *sra* in *Aβ42*-expressing flies exacerbated the rough-eye phenotype (H-J). By contrast, *sra* deficiency (E) partially rescued the rough-eye phenotype (K) as compared with that of *Aβ42*-expressing flies (H). The rough-eye phenotype induced by *sra* overexpression was rescued by *DIAP1* overexpression (F,L). Inset figures are high-magnification images. (M) The graph shows the relative eye size of each experimental group (Tukey–Kramer test, *n*≥9, *P<0.05, ***P<0.001). (N-P) Aβ42 levels do not change following expression of *sra*. (N,O) Aβ42 mRNA (N) and protein (O) in the larval eye discs of each group (N, Student's *t*-test, *n*=8; O, Student's *t*-test, *n*=7; NS, not significant). (P) Confocal images showing the presence of Aβ42 in larval eye discs of the indicated groups. More than 20 discs were observed for each group, and the representative images are shown. Magnification of the pictures, ×200. (Q) Representative images of Thioflavin-S staining in the brains of 20-day-old male flies. No prominent difference in staining was observed between brains of homozygous *Aβ42*-expressing flies with (*elav*>*Aβ42*+*sra^EY^*) or without (*elav*>*Aβ42*) *sra* overexpression. No signal was detected in the control (*elav-GAL4*). Magnification of the pictures, ×400. (R) Survival rates of pan-neuronal *Aβ42*-expressing flies with *sra* overexpression (*elav*>*Aβ42+sra^EY^*) during development. The effects of overexpressed *sra* (*elav>sra^EY^*) in the controls (*elav-GAL4*, *UAS-Aβ42*, *sra^EY^*) are also shown (Tukey–Kramer test, *n*≥250, *P<0.05, **P<0.01, ***P<0.001). (S) Effect of *sra* overexpression on the locomotor activity of pan-neuronal *Aβ42*-expressing flies. Climbing assay was performed using 10-day-old male flies (Tukey–Kramer test, *n*=100, ***P<0.001, NS, not significant). (T) Survival curve of pan-neuronal *Aβ42*-expressing male flies with *sra* overexpression (*elav*>*Aβ42+sra^EY^*). The lifespans of *sra*- (*elav>sra^EY^*) or *Aβ42*-(*elav*>*Aβ42*) expressing flies and control flies (*elav-GAL4*) are also presented (Kaplan–Meier estimator and log-rank test, *n*≥100). All data are expressed as mean±s.e.m.

Previous studies have reported that ectopic expression of *Aβ42* in *Drosophila* eyes resulted in a strong rough-eye phenotype, which is a useful marker for cytotoxicity (Hong et al., 2011, 2012). To study the role of *sra* in AD pathology, we examined the effect of *sra* overexpression on the *Aβ42*-induced rough-eye phenotype. Interestingly, upregulation of *sra* expression using *sra^EY07182^* or *UAS-sra* in the developing eyes of *Drosophila* resulted in a mild but prominent rough-eye phenotype (Fig. 2C,D) compared to that of the control (Fig. 2A,B,G). The expression levels of *sra* induced by *sra^EY07182^* were measured in the heads of neuronal *sra*-overexpressing flies (*elav*>*sra^EY^*) by real-time quantitative PCR (Fig. S2A), which confirmed that *sra* transcript levels were increased by approximately twofold compared to those of the control (*elav*-*GAL4* or *sra^EY^*), a similar degree to that shown in previous reports (Chang et al., 2003; Shaw and Chang, 2013). We also confirmed that the expression of a neighboring gene, *Bin1*, was not affected in *sra^EY07182^* flies (Fig. S2B).

In the next experiment, we examined the effect of *sra* overexpression in the *Aβ42*-induced rough-eye phenotype. As reported previously (Hong et al., 2011, 2012), ectopic expression of *Aβ42* in *Drosophila* eyes caused a small- and rough-eye phenotype (Fig. 2G,H,M). Interestingly, the small- and rough-eye phenotype of *Aβ42*-expressing flies was exacerbated by *sra* overexpression (Fig. 2C,D,H-J,M), suggesting that the elevated level of *sra* increased Aβ42 cytotoxicity. By contrast, a reduction in *sra* levels caused by *sra* deficiency rescued the *Aβ42*-induced phenotypes (Fig. 2E,H,K,M). The *sra*-induced rough-eye phenotype was completely rescued by *Drosophila inhibitor of apoptosis protein 1* (*DIAP1*), a caspase inhibitor, which suggests that *sra* overexpression induces apoptosis through caspase activation (Fig. 2C,F,L,M). Consistently, we found that *sra* overexpression induced cell death in the eye imaginal disc and increased *Aβ42*-induced cell death (Fig. S3). Next, we investigated whether *sra* altered Aβ42 expression and accumulation using real-time quantitative PCR, western blot analyses, immunohistochemistry and Thioflavin S staining. As shown in Fig. 2N-Q, Aβ42 expression and accumulation was not affected by altered *sra* expression levels, which suggests that the aggravated rough-eye phenotype induced by *sra* overexpression might not be due to alterations in Aβ42 accumulation. Consistent with the effect on the eye phenotype, upregulation of *sra* expression in the neuronal *Aβ42*-expressing flies decreased survival rates during development (Fig. 2R). Moreover, neuronal overexpression of *sra* in combination with *Aβ42* dramatically reduced both locomotor activity and the adult lifespan of *Aβ42*-expressing flies (Fig. 2S,T, and Table 1). Comparatively, flies with overexpression of *sra* alone showed normal climbing ability albeit with a slightly reduced lifespan (Fig. 2S,T, and Table 1). Taken together, these results suggest that increased *sra* expression alone can exert detrimental effects on both development and adult neuronal function in *Drosophila.* When combined with *Aβ42*, *sra* overexpression seems to enhance the cytotoxic effects associated with this gene product.

**Table 1.**
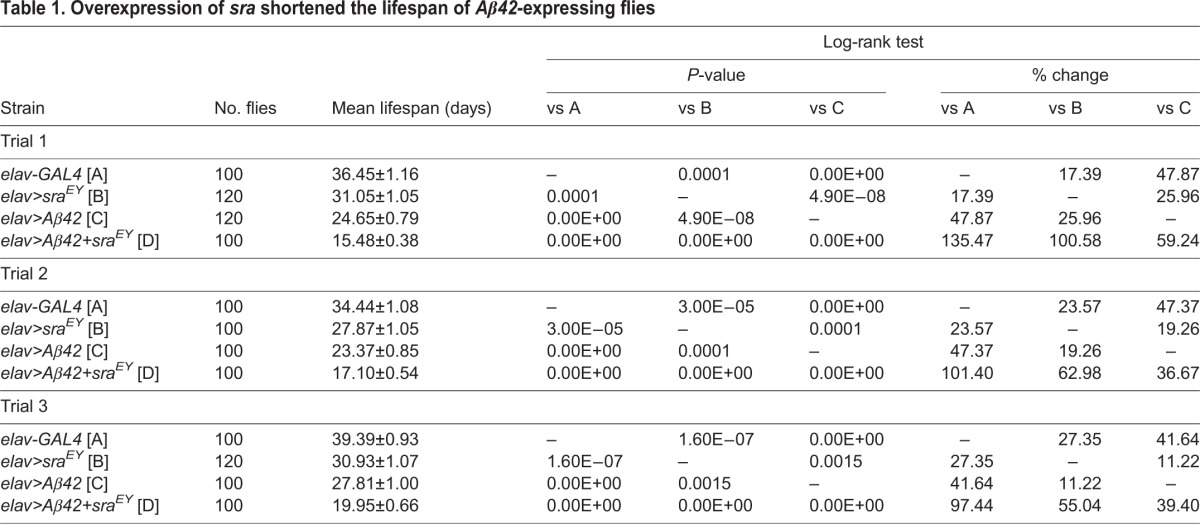
**Overexpression of *sra* shortened the lifespan of *Aβ42*-expressing flies**

Although Aβ42 is a processed product of APP, a previous study reported that *sra* delays neurodegeneration and ameliorates axonal transport defects induced by *APP* expression (Shaw and Chang, 2013). Therefore, we tested whether *sra* overexpression suppressed the phenotypes of *APP*-expressing flies. Interestingly, *sra* slightly rescued the rough-eye phenotype and increased the survival of *APP*-expressing flies (Fig. S4A-E).

### Overexpression of *sra* increased *Aβ42*-induced neuronal cell death

**Fig. 3.**
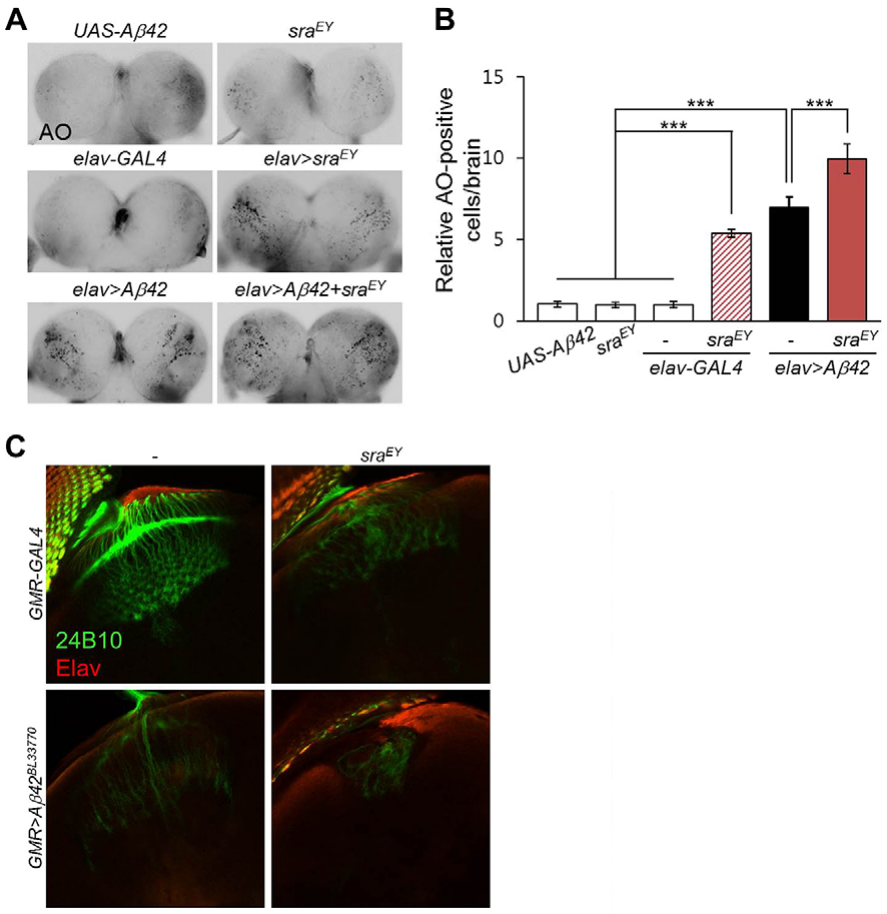
**Overexpression of *sra* induces neurodegeneration and aggravates *Aβ42*-induced phenotypes in *Aβ42*-expressing flies.** (A) Acridine orange (AO)-staining images of larval brains. (B) The graph shows the average number of AO-positive cells in the larval brains of each experimental group (Tukey–Kramer test, *n*=10, ***P<0.001). The data are expressed as mean±s.e.m. (C) Overexpression of *sra* increased *Aβ42*-induced defects in photoreceptor axon targeting. Photoreceptor axon projections in a late third-instar larval brain were stained with an anti-Chaoptin antibody (24B10). The anti-Elav antibody highlights whole neurons. Magnification of the pictures, ×400.

Because *sra* overexpression induced apoptosis in the developing eye, we investigated whether the elevated *sra* levels influenced *Aβ42*-induced neuronal cell death. To test this, we examined cell death in the larval brains of *sra*- or *Aβ42*-expressing flies using acridine orange (AO) staining. As shown in Fig. 3A,B, *sra* overexpression induced prominent cell death in the brain and further increased *Aβ42*-induced cell death. Next, we tested whether *sra* overexpression influences neurodegeneration of photoreceptor neurons in the larval brain and eye imaginal discs by immunohistochemistry using the anti-Chaoptin antibody (24B10). As expected, elevated *sra* levels greatly increased *Aβ42*-induced neurodegeneration and axon targeting defects in photoreceptor neurons (Fig. 3C, Fig. S5).

### Overexpression of *sra* increased the number of glial cells in the larval brain

**Fig. 4.**
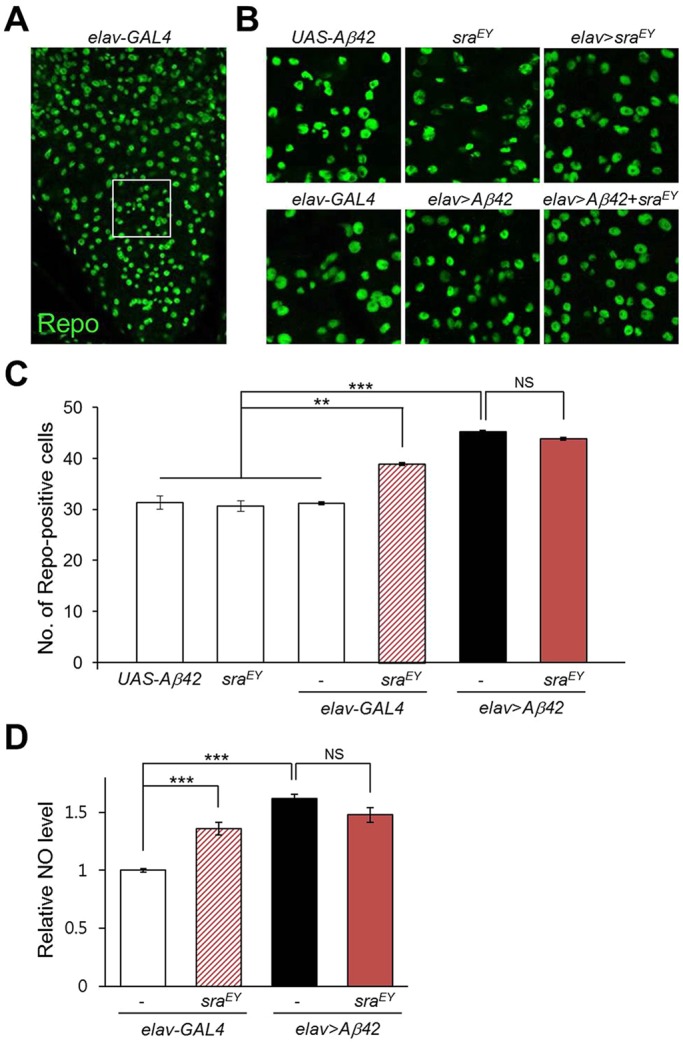
**Pan-neuronal overexpression of *sra* increases the number of glial cells and nitric oxide (NO) levels in *Drosophila* brains.** (A) Representative immunohistochemistry images of larval brain stained with an anti-Repo antibody. (B) Confocal images of the larval brains with indicated genotype corresponding to the white box in A. Magnification of the pictures: (A) ×200, (B) ×400. (C) The graph shows the number of Repo-positive cells (Tukey–Kramer test, *n*≥20, **P<0.01, ***P<0.001, NS, not significant). (D) Overexpression of *sra* or *Aβ42* increases NO levels in the adult fly head region (Tukey–Kramer test, *n*≥6, ***P<0.001, NS, not significant). All data are expressed as mean±s.e.m.

Previously, we found that ectopically expressed human *Aβ42* increased the number of glial cells in the larval brain as a result of neuronal damage (Park et al., 2013). Although *sra* expression did not alter *Aβ42* levels, we examined whether it still affected glial cell numbers. Upon immunostaining with antibodies against the glial-cell-specific Repo protein, *sra* overexpression alone in neurons increased the number of glial cells in the larval brain (Fig. 4A-C). Moreover, nitric oxide (NO) levels in the fly head region were also increased by *sra* overexpression, as in *Aβ42*-expressing flies, compared to that of the control (Fig. 4D). These observations might be explained by increased neuroinflammation possibly induced by *sra* overexpression, resulting in glial cell proliferation and subsequent harmful effects on neurons as well. Interestingly, however, elevated *sra* levels in the brain of *Aβ42*-expressing larvae did not further increase glial cell numbers (Fig. 4B,C) or NO levels (Fig. 4D), indicating that overexpressed *sra* and *Aβ42* might target identical pathway(s) to induce glial cell proliferation.

### Overexpression of *sra* altered hydrogen peroxide susceptibility, mitochondrial function, and anti-ROS protective pathways

**Fig. 5.**
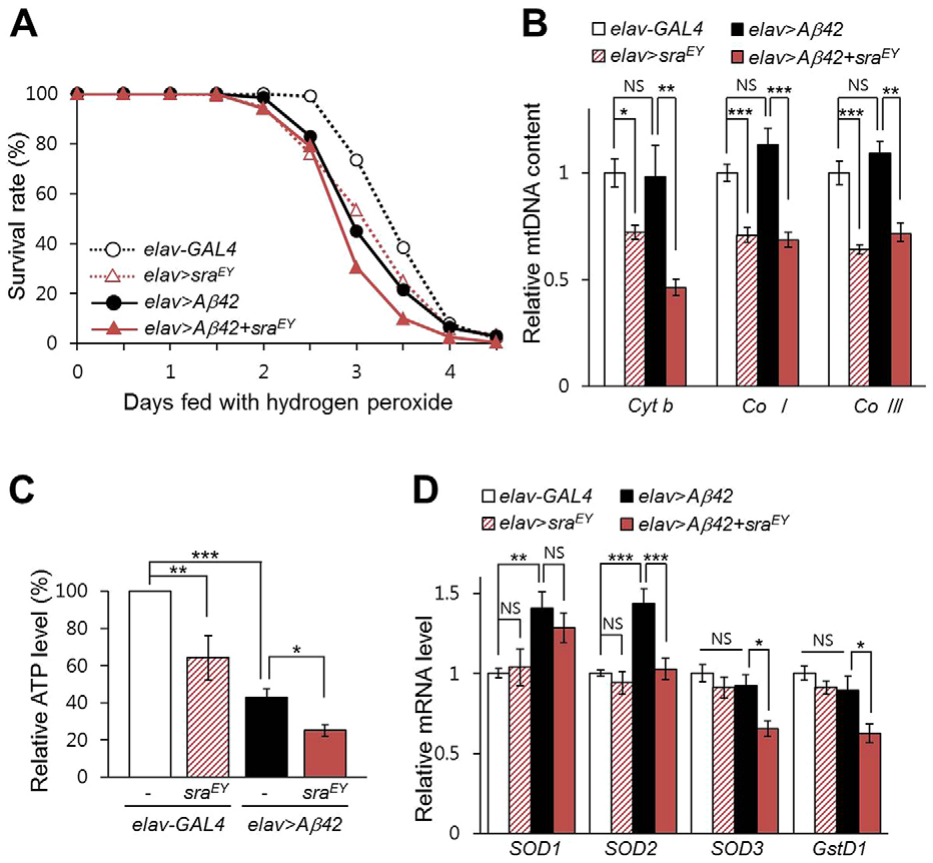
**Overexpression of *sra* increases oxidative stress susceptibility and induces mitochondrial dysfunction.** (A) Survival rates of *Aβ42*-expressing flies overexpressing *sra* under oxidative stress conditions (*n*=200). The Kaplan–Meier estimator and log-rank test was used to determine significant differences in survival rates of samples. *elav*-*GAL4* vs *elav*>*sra^EY^*: *P*=0.0001; *elav*-*GAL4* vs *elav*>*Aβ42*: *P*<0.0001; *elav*>*sra^EY^* vs *elav*>*Aβ42*+*sra^EY^*: *P*=0.0001; *elav*>*Aβ42* vs *elav*>*Aβ42*+*sra^EY^*: *P*=0.0004. (B) The relative levels of mtDNA were determined with real-time quantitative PCR using primers for *mitochondrial cytochrome b* (*Cyt b*) and *mitochondrial cytochrome c oxidase subunits I* and *III* (*Co I* and *Co III*). Tukey–Kramer test, *n*=9, *P<0.05, **P<0.01, ***P<0.001, NS, not significant. (C) Overexpression of *sra* decreased ATP levels in *Aβ42*-expressing flies. ATP levels were measured using a bioluminescent assay (Tukey–Kramer test, *n*≥4, *P<0.05, **P<0.05, ***P<0.001). (D) Relative mRNA levels of ROS response-associated genes were determined with real-time quantitative PCR using primers for *superoxide dismutase subunits 1*, *2* and *3* (*SOD1*, *SOD2* and *SOD3*) and *glutathione S transferase D1* (*GstD1*) (Tukey–Kramer test, *n*≥18, *P<0.05, **P<0.01, ***P<0.001, NS, not significant). All data are expressed as mean±s.e.m.

Because increased *sra* levels exacerbate *Aβ42*-induced neuronal impairment, we investigated the role of *sra* in *Aβ42*-associated pathogenesis. Increased oxidative stress is the most important pathophysiological phenomenon in AD (Markesbery, 1997); thus, we examined whether altering *sra* expression affected the susceptibility of flies to hydrogen peroxide. As shown in Fig. 5A, *sra* overexpression decreased the survival of *Aβ42-*expressing flies exposed to hydrogen peroxide, which suggests that elevated *sra* levels increased the susceptibility of *Aβ42* flies to oxidative stress. However, *sra* overexpression did not affect the susceptibility of *APP*-overexpressing flies to oxidative stress (Fig. S4F).

Because previous studies showed that both *sra* and *DSCR1* overexpression altered mitochondrial functions (Chang and Min, 2005; Ermak et al., 2012), we tested whether *sra* overexpression affects mtDNA levels. Although mtDNA levels were not altered by ectopic *Aβ42* expression, *sra* overexpression significantly decreased mtDNA levels in *Aβ42*-expressing flies (Fig. 5B). We also measured the ATP levels in the head of *sra-* or *Aβ42*-expressing flies (Fig. 5C). As expected, *sra* overexpression in neurons significantly reduced ATP levels (Fig. 5C). Interestingly, *Aβ42* expression also markedly reduced ATP levels (Fig. 5C), although it did not affect mtDNA levels, unlike *sra* overexpression, which suggests that Aβ42 alters mitochondrial functions via a different mechanism to that of Sra. Indeed, co-expression of both *sra* and *Aβ42* further decreased ATP levels compared to those with overexpression of *sra* or *Aβ42* alone (Fig. 5C).

We also tested whether *sra* overexpression affected anti-ROS protective pathways by measuring *SOD1*, *SOD2*, *SOD3* and *GstD1* mRNA levels in the head of *sra-* or *Aβ42*-expressing flies. As shown in Fig. 5D, none of the tested genes were affected by the overexpression of *sra* alone. In *Aβ42*-expressing flies, *SOD1* and *SOD2* expression were increased (Fig. 5D), even though these flies showed increased susceptibility to oxidative stress (Fig. 5A). This might be the result of a self-protecting mechanism of the cells against the increased levels of ROS. Interestingly, *SOD3* and *GstD1* expression were significantly reduced in the head of *sra-* and *Aβ42*-expressing (*elav*>*Aβ42*+*sra^EY^*) flies (Fig. 5D), although these expression levels were not affected by *Aβ42-* or *sra-*overexpression alone, which suggests that some of the detrimental effects of *sra* overexpression in *Aβ42*-expressing flies might be caused by the impairment of anti-ROS protective pathways associated with SOD3 and GstD1.

### Calcineurin inhibition deteriorated the phenotypes of *Aβ42*-expressing flies

**Fig. 6.**
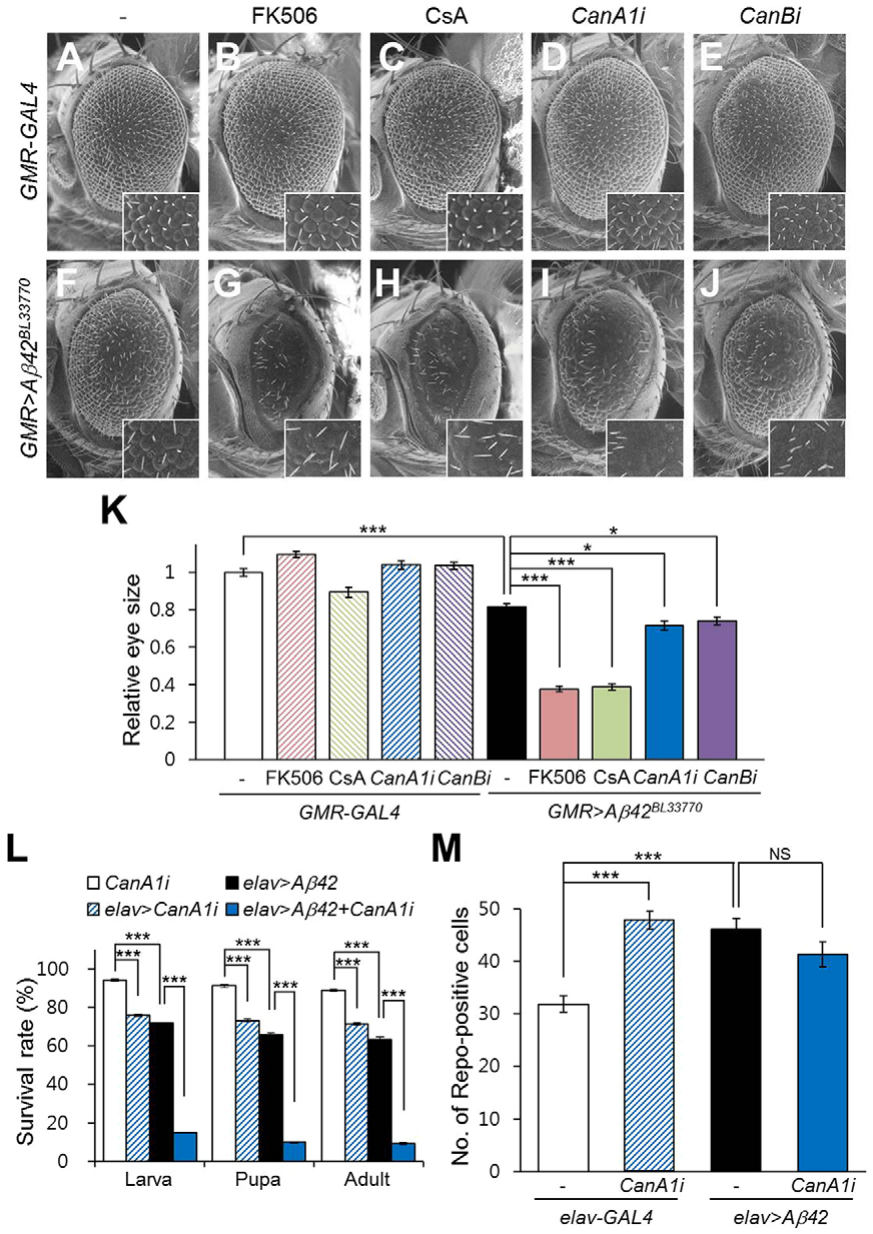
**The phenotypes of *Aβ42*-expressing flies are exacerbated by chemical calcineurin inhibitors or *calcineurin* RNAi.** (A-C,F-H) Representative images of developing eye phenotypes in DMSO-fed (A,F), FK506-fed (B,G) and CsA-fed (C,H) flies with (F,G,H) or without (A-C) *Aβ42* expression. (D,E,I,J) Representative images of developing eye phenotypes in flies expressing *calcineurin A1* RNAi (*CanA1i*) or *calcineurin B* RNAi (*CanBi*) with (I,J) or without (D,E) *Aβ42*. Inset figures are high-magnification images. (K) The graph shows the relative eye size in each experimental group (Tukey–Kramer test, *n*≥10, *P<0.05, ***P<0.001). (L) The effect of *CanA1* knockdown on the survival rate of *Aβ42*-expressing flies (Tukey–Kramer test, *n*≥350, ***P<0.001). (M) *CanA1* knockdown in neurons increased the number of glial cells in the larval brain. The graph shows the number of Repo-positive cells (Tukey–Kramer test, *n*≥15, ***P<0.001, NS, not significant). CsA, cyclosporin A.

Because the major function of DSCR1 is to inhibit calcineurin, we investigated whether chemical inhibitors of calcineurin mimic the effect of *sra* overexpression on *Aβ42-*induced cytotoxicity. To evaluate the effect of calcineurin inhibitors, the eye phenotype of *Aβ42*-expressing flies was examined after feeding with the calcineurin inhibitors FK506 and cyclosporin A (CsA). Because high-dose feeding (0.5 mM FK506 and 0.2 mM CsA) resulted in lethality, we used relatively low doses (50 µM FK506 and 20 µM CsA), and most flies survived to adulthood. The inhibitory effects of these compounds at these concentrations were confirmed by examining the rescue of hyperactivated calcineurin-induced wing phenotypes – the loss of wing veins and the reduction in wing size (Takeo et al., 2010) – following drug administration (Fig. S6). The eyes of control flies (*GMR-GAL4*) fed with low-dose FK506 were not obviously different from those of the unfed control, and control flies fed with CsA showed a very mild rough-eye phenotype (Fig. 6A-C). However, interestingly, drug administration to *Aβ42*-expressing flies at the same dose prominently exacerbated the small- and rough-eye phenotype induced by *Aβ42* overexpression (Fig. 6F-H,K).

We also tested the effects of genetic knockdown of *Drosophila* calcineurin using *calcineurin* RNA interference (RNAi) on the *Aβ42*-induced rough-eye phenotype. As expected, the rough-eye phenotype of *Aβ42*-expressing flies was exacerbated by the decreased levels of both calcineurin A and B (Fig. 6D-F,I-K). These results suggest that calcineurin activity is required to protect cells from *Aβ42* cytotoxicity.

Next, we examined whether alteration of calcineurin levels affects the Aβ42 phenotypes. Consistent with the results of *sra* overexpression, reduction of calcineurin levels using *CanA1* RNAi decreased fly survival rate and increased glial proliferation (Fig. 6L,M). Moreover, calcineurin reduction decreased the survival of *Aβ42*-expressing flies (Fig. 6L). However, interestingly, as with *sra* overexpression, *CanA1* RNAi did not increase *Aβ42*-induced glial cell proliferation (Fig. 6M).

## DISCUSSION

Almost all individuals with DS over 40 years of age show the characteristic neuropathology of AD (Lott and Head, 2005). Although overexpression of APP is the most probable cause of AD in individuals with DS, involvement of other genes has also been reported in its pathogenesis (Ermak et al., 2001; Kimura et al., 2007), among which is *DSCR1*. *DSCR1* is highly expressed in the AD brain (Ermak et al., 2001) and is implicated in various types of neuronal stresses linked to AD (Ermak and Davies, 2003; Belmont et al., 2008; Sun et al., 2014). Therefore, DSCR1 levels are expected to be closely associated with AD neuropathology. Here, we investigated the effect of overexpression of *sra*, a *Drosophila DSCR1* ortholog, on various *Aβ42*-induced phenotypes in *Drosophila* AD models. First, we observed that *sra* overexpression alone exerted detrimental effects on all observed phenotypes in comparison with those of control flies. These results are consistent with previous reports showing that upregulation of *sra* as well as mammalian *DSCR1* is detrimental for neurons (Ermak and Davies, 2003; Chang and Min, 2005; Keating et al., 2008; Ermak et al., 2011). Moreover, *sra* overexpression in the *Aβ42*-expressing flies exacerbated distinctive *Aβ42*-induced phenotypes. Thus, our data suggest that *sra* overexpression might not only produce detrimental effects on various cellular processes, but also boost Aβ42 cytotoxicity.

Recently, Shaw and Chang (2013) reported that *sra* upregulation provides a protective effect against *APP*-induced neurodegeneration and axonal transport defects. Regarding the *APP*-induced phenotypes, we also found similar protective effects of *sra*, which is inconsistent with the results from the experiments with *Aβ42*-expressing flies. Because Aβ42 is supposedly responsible for APP toxicity, it is interesting that Sra differentially affects Aβ42 and APP phenotypes. The discrepancy might be due to the differential involvement of Sra with the two molecules, APP and Aβ42. For example, Aβ42 is derived from proteolytic cleavage of APP, and Sra would protect APP toxicity by reducing the processing of APP. Because the proteolytic cleavage of APP is mediated by γ- and β-secretase [also known as beta-site APP-cleaving enzyme 1 (BACE1)] (Vassar et al., 1999; Yan et al., 1999), the regulation of these enzymes is an important mechanism underlying the pathogenesis of AD. Interestingly, a previous study demonstrated that calcineurin increased *BACE1* expression via nuclear factor of activated T cells 1 (NFAT1), resulting in increased Aβ generation in primary cortical cultures from Tg2576 mice (Cho et al., 2008). Therefore, overexpression of *DSCR1*, a calcineurin inhibitor, might reduce *BACE1* expression in neurons and thereby decrease Aβ generation, suggesting a negative role of calcineurin-dependent *BACE1* on the proteolytic cleavage of APP in *Drosophila*. However, it is currently uncertain whether this is the case because we did not detect any trace of Aβ42 production in human-*APP*-expressing flies, regardless of *sra* expression (Fig. S7).

Alternatively, the discrepancy between our data and those of Shaw and Chang (2013) might be due to differences in the physiological processes associated with APP or Aβ42. Previous studies have shown that overexpression of *APP* induced axonal transport defects in both flies and mice, independently of Aβ peptides (Gunawardena and Goldstein, 2001; Stokin et al., 2008). Moreover, similar axonal defects were found in early-stage human AD brains (Stokin et al., 2005). Interestingly, Shaw and Chang (2013) showed that *sra* overexpression decreased *APP*-induced neurodegeneration by ameliorating the axonal transport defects. They suggested that DSCR1 might delay the progression of AD in DS and that signaling pathways downstream of DSCR1 could be potential therapeutic targets for AD (Shaw and Chang, 2013). However, in the present study, we used different AD models in which *Aβ42* was expressed directly. In these models, axonal transport and processing of APP were bypassed, and the effects of Aβ42 were focused. Unlike the data with *APP*-expressing flies, we did not find any prominent alterations in Aβ42 accumulation and aggregation owing to *sra* overexpression, although the elevated *sra* levels altered the phenotypes of *Aβ42*-overexpressing flies. Therefore, we believe that *sra* might exert its detrimental effects by affecting the cellular events downstream of Aβ42, rather than by regulating Aβ42 accumulation. Aβ42 has been reported to exert its cytotoxicity by several other mechanisms, including mitochondrial dysfunction, oxidative stress induction and Ca^2+^ influx (Mattson, 2004; Bezprozvanny and Mattson, 2008), which are associated with DSCR1 function. These reports are supportive of our results showing that increased *sra* levels caused mitochondrial dysfunction and increased susceptibility to oxidative stress in *Aβ42*-expressing flies. Therefore, this additional effect of *sra* overexpression on Aβ42-induced neuronal impairment might be the result of synergy between DSCR1 and Aβ42 during the cytotoxic cellular events.

Although our findings clearly demonstrate the detrimental effects of *sra* overexpression on *Aβ42*-expressing flies, several limitations prevent the exact identification of this mechanism at present. Firstly, we did not completely exclude the possibility that upregulated *sra* might reduce the generation of toxic Aβ42 oligomers. Because Aβ oligomers are important in AD pathology (Wirths et al., 2004), and Aβ oligomers are generated in *Drosophila* AD models (Iijima et al., 2004), further study is needed to clarify whether *sra* overexpression affects the generation of toxic oligomers. Second, it is also possible that the cytotoxic effects of Aβ42 in *Drosophila* might differ from human models in some respects. For example, unlike humans with AD or DS, *Aβ42*-expressing flies showed evidence of developmental problems. The relevance of *Aβ42*-expressing fly models needs to be verified in detail, especially with regard to developmental phenotypes.

Hyperactivated calcineurin, a calcium-activated phosphatase, is implicated in neuronal cell death, inflammation and plasticity (Reese and Taglialatela, 2010, 2011). Consequently, calcineurin inhibitors are expected to produce beneficial effects against AD neuropathology (Agostinho et al., 2008; Dineley et al., 2010; Brait et al., 2012; Sobrado et al., 2012; Shaw and Chang, 2013). However, several studies suggest that calcineurin might have a protective effect during the pathogenesis of AD or AD-related pathways. First, pre-treatment with calcineurin inhibitors significantly increases neuronal death induced by hydrogen peroxide (Porta et al., 2007), which suggests that calcineurin activity might be protective following oxidative stress. Secondly, calcineurin is implicated in the regulation of tau phosphorylation, hyperphosphorylation of which is one of the pathological signatures of AD (Poppek et al., 2006; Lloret et al., 2011). Thirdly, calcineurin exhibits an inhibitory role against epidermal growth factor receptor signaling during *Drosophila* development (Sullivan and Rubin, 2002), which was reported as a preferred target for treating Aβ-induced memory loss in both flies and mice (Wang et al., 2012). Consistently, in the present study, we demonstrated that treatment with calcineurin inhibitors or calcineurin knockdown exacerbated the *Aβ42*-induced rough-eye phenotype, indicating that calcineurin activity might play a protective role against Aβ42 cytotoxicity. Therefore, the inhibition of calcineurin activity could be responsible for the harmful effects of *sra* overexpression in this phenotype.

Because Aβ42 accumulation underlies AD pathology (Hardy and Selkoe, 2002) and is found in DS brains (Teller et al., 1996), our study suggests that increased DSCR1 expression in DS brains might influence rapid AD progression in the presence of Aβ42 neurotoxicity. According to our findings, it is likely that increased *DSCR1* expression in DS brains might contribute to AD progression via two different modes depending on the presence of Aβ42. In the absence of Aβ42, increased *DSCR1* expression might protect neurons by reducing APP-induced axonal transport defects that occur prior to APP processing. However, once Aβ42 is produced from APP, DSCR1 might exacerbate the harmful effects of Aβ42 to promote neurodegeneration in the DS brain. In the brains of most individuals with DS, a substantial amount of Aβ42 is normally present (Masters et al., 1985; Teller et al., 1996; Lott and Head, 2005). Therefore, a protective role of DSCR1 to counteract APP-induced neuronal damage might be very limited in the majority of DS cases. Accordingly, the increased expression of DSCR1 as seen in most DS brains would play a negative role on AD-related neuropathology.

In summary, we demonstrated that upregulation of *sra* expression or downregulation of calcineurin activity results in detrimental effects on *Drosophila* development. Moreover, these alterations in *sra* or *calcineurin* expression exacerbate most of the examined Aβ42-induced phenotypes. Therefore, our data indicate that chronic overexpression of *DSCR1* is detrimental to Aβ42-induced neurotoxicity, and that increased expression of *DSCR1* in the brain of individuals with DS or AD might exacerbate AD pathogenesis.

## MATERIALS AND METHODS

### *Drosophila* strains

*embryonic lethal abnormal vision* (*elav*)-*GAL4* (pan-neuronal driver), *glass multimer reporter* (*GMR*)-*GAL4* (eye driver), *en2.4-GAL4* (posterior compartment of imaginal discs driver), *UAS-2×enhanced green fluorescent protein* (*EGFP*) (BL6874), *sra^EY07182^* (BL15991), *UAS*-*CanA1 RNAi* (BL25850), *UAS*-*CanB RNAi* (BL27307), *UAS-Drosophila inhibitor of apoptosis protein 1* (*DIAP1*) (BL6657), *UAS-Aβ42^BL33770^* (BL33770; a Bloomington *Drosophila* Stock Center version of *UAS-Aβ42*) and *UAS-APP-N-myc* (BL6700) were obtained from the Bloomington *Drosophila* Stock Center. *UAS-sra*, *UAS-CanA^act^* and *sra^KO^* were provided by Dr Toshiro Aigaki (Tokyo Metropolitan University, Japan). *UAS-Aβ42* was provided by Dr Mary Konsolaki (Rutgers University, USA). To isogenize the genetic background, *elav-GAL4*, *UAS-Aβ42*, *UAS-Aβ42^BL33770^*, *sra^EY07182^* and *UAS*-*CanA1 RNAi* were backcrossed with *w^1118^* six times. Because the *UAS-Aβ42^BL33770^* construct contains an α-tubulin 3′ UTR, which provides stability to the *Aβ42* mRNA produced from the Scer\UAS regulatory sequences (Ollmann et al., 2000), it exerts stronger cytotoxic effects than the *UAS-Aβ42* construct. Therefore, we used the *UAS-Aβ42^BL33770^* strain to analyze the cytotoxic effects of Aβ42 on fly eye development. However, because most *elav*>*Aβ42^BL33770^* flies die during embryogenesis, the *UAS-Aβ42* strain was used to investigate the effect of *Aβ42* expression in the neurons of larvae and adult flies. The genotypes of flies used in this study are denoted in Table S1.

### Generation of *sra-GAL4*

To generate the *sra-GAL4* transgenic fly, 960 bp of the *sra* promoter region was amplified by PCR from *w^1118^* genomic DNA and sub-cloned into the *Bgl*II/*Kpn*I site of the pPTGAL vector (Sharma et al., 2002). The construct was confirmed by sequencing. The transgenic lines were established in a *w^1118^* background. The primer sequences used were (*Bgl*II and *Kpn*I linker sequences are shown in italics) as follows: 5′-GA*AGATCT*CAGCTCGTAGTTCGTCTTAC-3′ (forward) and 5′-GG*GGTACC*GACGATTGTCATGCCAGG-3′ (reverse).

### Immunohistochemistry

For immunohistochemistry with larval eye imaginal discs or larval brains, samples were fixed in 4% paraformaldehyde for 4 min and washed four times with phosphate-buffered saline (PBS) containing 0.1% Triton X-100 (PBST). Tissues were blocked with 2% normal goat serum (NGS) in PBST and incubated overnight with mouse anti-Repo [1:10; 8D12, Developmental Studies Hybridoma Bank (DSHB), Iowa City, IA, USA], mouse anti-Chaoptin (1:200; 24B10, DSHB, Iowa City, IA, USA), mouse anti-Aβ42 (1:200; DE2B4, sc-58508, Santa Cruz Biotechnology, Dallas, TX, USA), rat anti-Elav (1:200; 7E8A10, DSHB, Iowa City, IA, USA) or rabbit anti-Sra (1:50; a gift from Dr Toshiro Aigaki, Tokyo Metropolitan University, Japan) antibodies at 4°C. The samples were then incubated with Alexa-Fluor-555-labeled anti-mouse, Alexa-Fluor-488-labeled anti-mouse, Alexa-Fluor-555-labeled anti-rabbit or Alexa-Fluor-594-labeled anti-rat secondary antibodies (1:200; Invitrogen, Carlsbad, CA, USA) for 1 h. For immunohistochemistry with adult brains, whole bodies of 3- to 5-day-old male flies were fixed in 4% paraformaldehyde containing 0.5% Triton X-100 at room temperature for 3 h. Whole brains were dissected out, blocked with 5% NGS and 2% bovine serum albumin in PBS containing 0.5% Triton X-100 for 3 h. They were then stained with mouse anti-Fasciclin II (1:200; 1D4, DSHB, Iowa City, IA, USA) or rabbit anti-Sra (1:50; a gift from Dr Toshiro Aigaki) at 4°C for 48 h. After washing four times with PBS containing 0.5% Triton X-100, samples were incubated at 4°C overnight with Alexa-Fluor-555-labeled anti-mouse or anti-rabbit antibodies (1:200; Invitrogen, Carlsbad, CA, USA). Samples were mounted with Vectashield mounting media (Vector Laboratories, Burlingame, CA, USA).

### Thioflavin S staining

Thioflavin S staining was performed as previously reported (Iijima et al., 2004). Adult fly brains were fixed in 4% paraformaldehyde containing 0.5% Triton X-100 for 3 h and washed three times with PBST. The samples were permeabilized and incubated in 50% ethanol containing 0.125% Thioflavin S (Sigma-Aldrich, St Louis, MO, USA) overnight at 4°C. After washing in 50% ethanol and PBST, brains were observed by confocal microscopy.

### Counting Repo-positive cells

After immunohistochemistry with an anti-Repo antibody, confocal images of larval brains were obtained. We counted the number of Repo-positive cells located in a 100×100 µm square of the dorsal region of a ventral ganglion. The mean number of Repo-positive cells per brain region of each indicated genotype was determined.

### Preparation of RNA and real-time quantitative PCR

Total RNA was isolated from the *Drosophila* heads with TRIzol (Invitrogen, Carlsbad, CA, USA). For the real-time quantitative PCR, cDNA was synthesized using a Maxime kit (iNtRON Biotechnology, Korea) and real-time quantitative PCR was performed using SYBR Green PCR Master Mix (Applied Biosystems, Carlsbad, CA, USA). Quantification was performed using the ‘delta-delta Ct’ method to normalize to *tubulin* transcript levels and to a control. The relative level of *sra*, *Aβ42*, *SOD1*, *SOD2*, *SOD3*, *GstD1* or *Bin1* mRNA to *tubulin* mRNA was statistically analyzed by Tukey–Kramer test. The following primer pairs were used (forward and reverse): *sra* (5′-CACGCCATGGAGGAGTTATT-3′ and 5′-TACTGGTGCAGCTTGATTCG-3′), *tubulin* (5′-TGTCGCGTGTGAAACACTTC-3′ and 5′-AGCAGGCGTTTCCAATCTG-3′), *Aβ42* (5′-TCCGACATGACTCAGGATATG-3′ and 5′-GCTATGACAACACCGCCCA-3′), *SOD1* (5′-CAACATCACCGACTCCAAGA-3′ and 5′-TTGACTTGCTCAGCTCGTGT-3′), *SOD2* (5′-ATCGAGTCGCAGTGGAAGAG-3′ and 5′-CAGTTTGCCCGACTTCTTGT-3′), *SOD3* (5′-AGCTGGAGGGATTGAAGGAG-3′ and 5′-GGGGCCACCGTGATCAAC-3′), *GstD1* (5′-CATCGCGAGTTTCACAACAGA-3′ and 5′-CTGTCCCTCCAGGAAGGTGTT-3′) and *Bin1* (5′-ACTTCCAGATGCGCGAAATC-3′ and 5′-GCGGAGTAATCGAGATGTCC-3′).

### Western blot analysis

For western blot analyses, adult fly heads were homogenized in 2× Laemmli sample buffer, and the lysates were separated by SDS-PAGE. Membranes were blocked with 5% non-fat dry milk and probed with anti-Sra (1:1000; a gift from Dr Toshiro Aigaki), anti-Aβ42 (1:2000; 6E10, Covance, UK), anti-Myc (1:2000; Cell Signaling Technology, Beverly, MA, USA) or anti-Actin (1:2000; JLA20, DSHB, Iowa City, IA, USA) antibodies. Western blot analyses were performed using standard procedures and horseradish-peroxidase-conjugated secondary antibodies (1:2000; Cell Signaling Technology, Beverly, MA, USA).

### Analysis of *Drosophila* development

Fifty age-matched embryos of each genotype were plated on grape-juice agar plates. After incubation at 25°C, the hatched larvae were transferred to vials with standard cornmeal media and aged at 25°C. The numbers of pupae and adult flies were recorded. The experiments were repeated at least five times. All data are expressed as mean±s.e.m. The data were quantitatively analyzed by Tukey–Kramer test.

### Climbing assay

The climbing assay was carried out as previously described (Hwang et al., 2013) with minor modifications. After collecting ten male flies in the climbing ability test vial, flies were incubated for 1 h at room temperature for environmental adaptation. Using the negative geotropism of *Drosophila*, we dropped the flies down to the bottom and counted the number of flies that climbed to the top of the vial within 15 s. Ten trials were conducted for each group. The experiment was repeated at least ten times with independently derived transgenic lines. Therefore, a total of 100 flies were analyzed for each group. Climbing scores (the ratio of the number of flies that climbed to the top against the total number of flies) were obtained for each group, and the mean climbing scores for ten repeated tests were compared.

### Longevity assay

To measure the adult lifespan, flies were maintained at 25°C on standard cornmeal agar medium. Twenty male flies were kept in one vial. More than five vials (>100 flies) were tested per group. The flies were transferred to fresh vials, and the number of living flies was counted every 3 days. The experiment was repeated three times with independently derived transgenic lines.

### Detection of nitric oxide levels

The 20 heads of 3-day-old male flies were prepared in homogenizing buffer (0.1 M phosphate buffer at pH 7.4, 25 mM KCl) on ice. After homogenization, samples were centrifuged at 10,000 ***g*** for 10 min at 4°C, and supernatants were collected. Greiss reagent (Sigma-Aldrich, St Louis, MO, USA) was added to the samples in a 1:1 ratio, and samples were incubated for 15 min at 25°C. Nitrite levels were measured using a NanoDrop spectrophotometer at 550 nm, and the relative nitrite levels of each group were statistically analyzed by Tukey–Kramer test.

### Acridine orange staining

Acridine orange (AO) staining was performed as reported previously (Hong et al., 2012). Larval brains and eye imaginal discs of stage L3 larvae were dissected in PBS. Then, the brains or discs were incubated for 5 min in 1.6×10^−6^ M AO (Sigma-Aldrich, St Louis, MO, USA) and rinsed two times for 5 min in PBS. The samples were subsequently observed under an Axiophot2 fluorescence microscope (Carl Zeiss, Jena, Germany).

### Oxidative stress test

The susceptibility to oxidative stress and its effects on the survival of each genotype were estimated with hydrogen peroxide. Two hundred flies of each genotype were starved for 6 h and transferred to vials with 5% sucrose solution containing 1% hydrogen peroxide. The number of live flies was recorded every 12 h.

### Mitochondrial DNA PCR

Mitochondrial DNA (mtDNA) from the *Drosophila* head was extracted using a ReliaPrep™ gDNA Tissue Miniprep System (Promega, Fitchburg, WI, USA). Each reaction was performed in a final volume of 20 μl using 20 ng of DNA. Real-time quantitative PCR was performed using SYBR Green PCR Master Mix (Applied Biosystems, Carlsbad, CA, USA) and mtDNA gene-specific primers. These primers selectively amplify the sense or antisense mitochondrial transcripts. Quantification was performed using the ‘delta-delta Ct’ method to normalize to *actin* transcript levels and to a control. Real-time quantitative PCR was performed using the following primer pairs (forward and reverse): *mitochondrial cytochrome c oxidase subunit I* (*Co I*; 5′-CAGGATGAACTGTTTATCCACCTTT-3′ and 5′-CCTGCTAAATGTAGAGAAAAAATAG-3′), *mitochondrial cytochrome c oxidase subunit III* (*Co III*; 5′-CAGACTCAATTTATGGATCAACATT-3′ and 5′-AAGTTGTTCCGATTAATACATGAAT-3′), *mitochondrial cytochrome b* (*Cyt b*; 5′-TTAATCATATTTGTCGAGACGTT-3′ and 5′-AATGATGCACCGTTAGCAT-3′) and *actin* (5′-CACCGGTATCGTTCTGGACT-3′ and 5′-GCGGTGGTGGTGAAAGAGTA-3′). Each experiment was repeated at least nine times (*n*=9). The relative level of *Co I*, *Co III* or *Cyt b* DNA to *actin* DNA was statistically analyzed by Tukey–Kramer test.

### ATP assay

ATP assays were conducted as described (Pogson et al., 2014) with some modifications. Briefly, ten heads of 3- to 5-day-old male flies were homogenized in 100 µl extraction buffer (6 M guanidine-HCl, 100 mM Tris, 4 mM EDTA, pH 7.8). After homogenization, samples were frozen in liquid nitrogen, followed by boiling for 5 min. The samples were centrifuged at 18,400 ***g*** for 3 min at 4°C, and supernatants were diluted (1:10) with extraction buffer and mixed with a luminescent solution (CellTiter-Glo Luminescent Cell Viability Assay, Promega, Fitchburg, WI, USA). Luminescence was measured on a Veritas™ Microplate Luminometer (Promega, Fitchburg, WI, USA). Relative ATP levels were calculated by dividing the luminescence by the concentration of the control. The relative ATP levels of each group were statistically analyzed by Tukey–Kramer test.

### Calcineurin inhibitor treatment

Fifty embryos of each genotype were reared in standard plastic vials with media containing 0.1% (v/v) DMSO (control), 50 µM FK506 (Sigma-Aldrich, St Louis, MO, USA) dissolved in 0.1% DMSO, or 20 µM cyclosporin A (Sigma-Aldrich, St Louis, MO, USA) dissolved in 0.1% DMSO.

### Statistics

In all experiments, data was quantitatively analyzed for statistical significance using either a Student's *t*-test (two-tailed) or a one-way ANOVA followed by a Tukey–Kramer multiple comparisons test. The Student's *t*-test was applied for comparisons of two groups. GraphPad Prism, Version 5.0 (GraphPad Software, San Diego, CA, USA) software was used and differences were significant when *P*<0.05. Western blotting data was assessed using MultiGauge version 3.1 (Fuji, Japan) software and converted into ratios of band intensity relative to the controls. Eye size was quantified using ImageJ software (National Institutes of Health, Bethesda, MD, USA). The Kaplan–Meier estimator and the log rank test were conducted on the cumulative survival under oxidative stress conditions and lifespan data to determine whether each condition had any effect on the longevity of individuals by using Online Application for the Survival Analysis of Lifespan Assays (http://sbi.postech.ac.kr/oasis).
